# Delayed Antiviral Immune Responses in Severe Acute Respiratory Syndrome Coronavirus Infected Pregnant Mice

**DOI:** 10.3389/fmicb.2021.806902

**Published:** 2022-01-21

**Authors:** Guohua Zhu, Shujuan Du, Yuyan Wang, Xixi Huang, Gaowei Hu, Xin Lu, Dajin Li, Yizhun Zhu, Di Qu, Qiliang Cai, Lu Liu, Meirong Du

**Affiliations:** ^1^Laboratory for Reproductive Immunology, NHC Key Lab of Reproduction Regulation (Shanghai Institute of Planned Parenthood Research), Shanghai Key Laboratory of Female Reproductive Endocrine-Related Diseases, Hospital of Obstetrics and Gynecology, Shanghai Medical College, Fudan University, Shanghai, China; ^2^MOE & NHC & CAMS Key Laboratory of Medical Molecular Virology, Department of Medical Microbiology and Parasitology, Shanghai Institute of Infectious Diseases and Biosecurity, School of Basic Medicine, Shanghai Medical College, Fudan University, Shanghai, China; ^3^Shanghai Key Laboratory of Bioactive Small Molecules, Department of Pharmacology, School of Pharmacy, Fudan University, Shanghai, China; ^4^State Key Laboratory of Quality Research in Chinese Medicine and School of Pharmacy, Macau University of Science and Technology, Taipa, Macau SAR, China; ^5^Department of Obstetrics and Gynecology, Guangzhou First People’s Hospital, School of Medicine, South China University of Technology, Guangzhou, China

**Keywords:** immune responses, SARS-CoV-2, fertility, pregnancy, T cell activation

## Abstract

Sex differences in immune responses had been reported to correlate with different symptoms and mortality in the disease course of coronavirus disease 2019 (COVID-19). However, whether severe acute respiratory syndrome coronavirus (SARS-CoV-2) infection interferes with females’ fertility and causes different symptoms among pregnant and non-pregnant females remains unknown. Here, we examined the differences in viral loads, SARS-CoV-2-specific antibody titers, proinflammatory cytokines, and levels of T cell activation after SARS-CoV-2 sub-lethal infection between pregnant and non-pregnant human Angiotensin-Converting Enzyme II (ACE2) transgenic mouse models. Both mice showed elevated levels of viral loads in the lung at 4 days post-infection (dpi). However, viral loads in the pregnant group remained elevated at 7 dpi while decreased in the non-pregnant group. Consistent with viral loads, increased production of proinflammatory cytokines was detected from the pregnant group, and the IgM or SARS-CoV-2-specific IgG antibody in serum of pregnant mice featured delayed elevation compared with non-pregnant mice. Moreover, by accessing kinetics of activation marker expression of peripheral T cells after infection, a lower level of CD8^+^ T cell activation was observed in pregnant mice, further demonstrating the difference of immune-response between pregnant and non-pregnant mice. Although vertical transmission did not occur as SARS-CoV-2 RNA was absent in the uterus and fetus from the infected pregnant mice, a lower pregnancy rate was observed when the mice were infected before embryo implantation after mating, indicating that SARS-CoV-2 infection may interfere with mice’s fertility at a specific time window. In summary, pregnant mice bear a weaker ability to eliminate the SARS-CoV-2 virus than non-pregnant mice, which was correlated with lower levels of antibody production and T cell activation.

## Introduction

Epidemiologic investigations indicate that pregnancy is associated with a higher risk of disease severeness and mortality after viral infections, such as influenza, Ebola, and Lassa fever (Silasi et al., 2015). Viral infection is also a major cause of adverse pregnancy outcomes such as spontaneous abortion and mother-to-child vertical transmission, resulting in serious consequences, such as congenital viral syndromes, intrauterine growth restriction, still-births, and fetal distress and loss. Investigating the potential risks of viral infections during pregnancy is essential for taking appropriate clinical interventions.

It has been 2 years since the global Coronavirus disease 2019 (COVID-19) pandemic outbreak. As of September, 2021, the number of patients confirmed to have COVID-19 has exceeded 220 million in 191 countries, including pregnant women (World-Health-Organization, 2021). Severe acute respiratory syndrome coronavirus 2 (SARS-CoV-2) is the pathogen responsible for the COVID-19 (Ren et al., 2020; Harvey et al., 2021). However, most pathogens that cause infections in pregnant mothers cannot reach the fetus, largely due to the potent protective mechanisms provided by placental cells, including syncytiotrophoblasts (Parry et al., 1997; Koi et al., 2002; Arora et al., 2017). However, some of the viruses, such as rubella virus, herpesvirus (HSV), cytomegalovirus (CMV), and Zika virus (ZIKV; Stegmann and Carey, 2002; Coyne and Lazear, 2016), among others, are capable of crossing the maternal-fetal barriers and infecting the fetus. Whether SARS-CoV-2 can cross the placenta barrier and cause adverse outcomes is still under debate (Diriba et al., 2020; Edlow et al., 2020; Schwartz, 2020; Damar Çakırca et al., 2021; Vouga et al., 2021). It has been shown that SARS-CoV-2 binds and infects host cells by utilizing the membrane-bound Angiotensin-Converting Enzyme II (ACE2) along with the viral S protein proteases, which is a key determinant of SARS-CoV-2 infection. Building upon single-cell RNA-sequencing data, researchers found that human placental cells express mRNA for SARS-CoV-2 receptors throughout pregnancy (Ashary et al., 2020).

Moreover, the SARS-CoV-2 virions had been found in the placenta of SARS-CoV-2 infected patients, which was complicated by severe preeclampsia and placental abruption (Hosier et al., 2020). The diffuse pathological changes in the placenta could be more likely associated with poor fetal outcomes rather than fetus infection (Bouachba et al., 2021; Cribiù et al., 2021). Some investigations revealed that the IgM or SARS-CoV-2 RNA had been detected in newborn infants (Juan et al., 2020; Song et al., 2021). Recently, a case was reported that SARS-CoV-2 had been found in the amniotic fluid and umbilical cord blood (Piñana et al., 2021). In summary, limited studies suggest the possibility of vertical transmission through the placental barrier. Whether vertical transmission would happen in the SARS-CoV-2 infection pregnant was still unclear.

Sex differences in immune responses had been reported to correlate with different symptoms and mortality in the disease course of COVID-19 (Meng et al., 2020; Takahashi et al., 2020; Vahidy et al., 2021). Researchers have found that the females had less-lethal clinical complications or stronger immune reactions in peripheral blood than males. Females undergo significant physiological changes during pregnancy, affecting the immune system, cardiopulmonary system, and coagulation. It has been reported that pregnant women are at greater risk of complications and severe disease from infection with other coronaviruses, including SARS and Middle Eastern Respiratory Syndrome (MERS; Wong et al., 2004; Di Mascio et al., 2020). Thus, they are identified as a vulnerable group and more likely to be intolerant to the SARS-CoV-2 infection.

Furthermore, infection is one of the important factors that may contribute to immune imbalance that finally result in implantation or pregnancy failure (Romero et al., 2004; Larsen et al., 2013). However, negligible information about health effects of SARS-CoV-2 infection is available for pregnant women. To gain an insight into the pathogenesis of SARS-CoV-2 infection during pregnancy, we utilized the human ACE2 (hACE2) transgenic mice to compare the outcome between non-pregnant and pregnant mice after SARS-CoV-2 infection.

## Materials and Methods

### Mice Model

**Figure 1:**
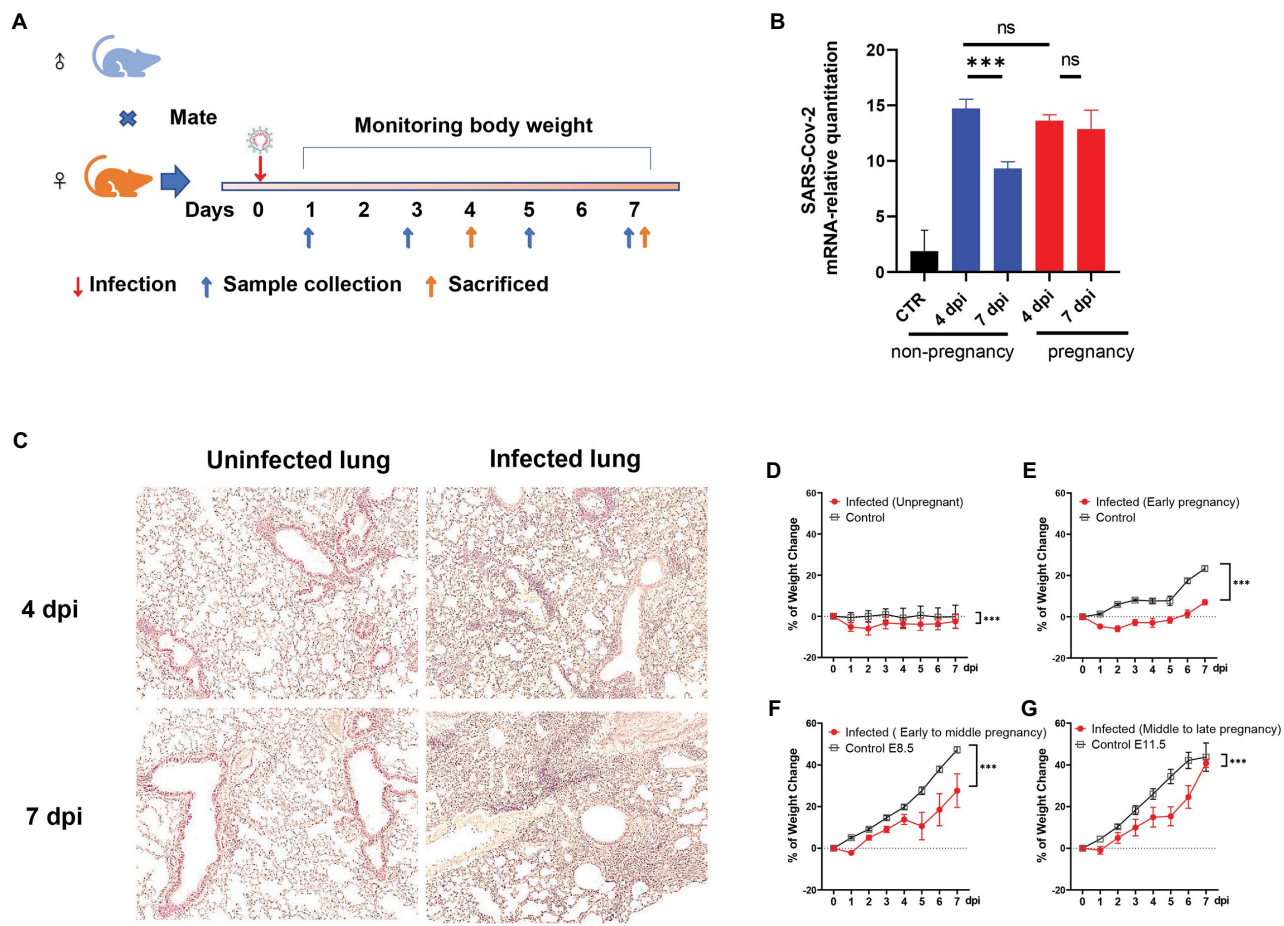
The establishment of the severe acute respiratory syndrome coronavirus (SARS-CoV-2) infected pregnant mice model. **(A)** The protocol of experiments. **(B)** The mRNA-relative quantitation of SARS-CoV-2 N expression in pulmonary tissues showed the difference in virus load between non-pregnant and pregnant during 4–7 days post-infection (dpi). CTR, control. **(C)** The pathological changes of the infected lung in hematoxylin and eosin (HE). **(D–G)** Weight changes. After being infected by virus or mock, the weights of the mice were recorded up to 7 dpi. Data were expressed as a percent of change from the baseline body weight (X ± SE, **D**) *n* = 13 for infected unpregnant, *n* = 25 for mock control; **(E)** *n* = 5 for infected (Early pregnant, started from E3.5 ± 1), *n* = 5 for pregnant control; **(F)** *n* = 8 for infected (Early to middle pregnant, started from E7.5 ± 1), *n* = 5 for pregnant control; and **(G)** *n* = 6 for infected (middle to late pregnant, started from E12.5 ± 1), *n* = 6 for pregnant control. Two-way ANOVA was used for time-related curve data here, while the Unpaired *t*-test or Mann-Whitney test was used for two groups compare. ns *p* > 0.05, ^***^p < 0.001.

The 6–8 weeks old transgenic hACE2-Chimera female mice (Cat: T037630, C57BL/6) and wild-type (WT) male mice (BALB/c) were purchased from the GemPharmaetch (Nanjing, China). The transgenic hACE2-Chimera female mice were generated *via* the CRISPR/Cas9 system, which had been used to explore the infectivity in coinfection in both influenza A and SARS-CoV-2 mice models (Ma et al., 2020). Here, the hACE2 expression in the uterus of transgenic mice had been verified by PCR. Gapdh primer was used for internal reference. The transgenic hACE2-chimera female mice were mated with WT BALB/c male mice. Both plugged female mice (*n* = 46) and unmated mice (*n* = 10) were anesthetized and intranasally infected with 50 μl (5 × 10^3^ PFU) of SARS-CoV-2 (nCoV-SH01-P7) deriving from the alveolar lavage fluid of COVID-19 patients. PBS (mock)-infected mice were used as controls. The weights of mice were monitored daily. Half of the pre-infected mice in each group were sacrificed at 4 days post-infection (dpi), and the others handled at 7 dpi. Dissections can confirm the actual state of pregnancy. Meanwhile, the blood samples were collected from the retro-orbital plexus at 1, 3, 5, and 7 dpi (Figure 1A). Subsequently, different standard operations were applied to inactivate SARS-CoV-2 virus, such as TRIzol lysis, heat inactivation, or 4% paraformaldehyde fixation (Patterson et al., 2020). All the infected mice were housed and infected at the biosafety level-3 (BSL-3) laboratory of Fudan University.

### RNA Extraction and Real-Time Quantitative PCR

Tissues were homogenized using a BeadBlaster homogenizer (JingXing Company, Shanghai) in 1 ml of TRIzol (Takara, Beijing, China). The virus RNA was extracted using SteadyPure Virus DNA/RNA Extraction Kit (Cat: AG21021) from Accurate Biology (Hunan, China) and then quantified by NanoDrop™ (Thermo Fisher, United States). The primer and probe sequence for SARS-CoV-2 nucleocapsid (N) transcripts provided by the Chinese Center for Disease Control and Prevention were used. Gapdh was used as the internal reference. The one-step probe Real-Time Quantitative PCR (RT-PCR) kit (Cat: AG11708, Accurate Biology, Hunan, China) was used to measure the viral load. Each reaction was performed in triplicate. Reverse transcription for extracted RNA of uterus tissue was performed with the Reverse Transcription System (Cat: AG11706, Accurate Biology, Hunan, China). Inflammatory cytokine in the non-pregnant mice’s uterus was detected by the SYBR green RT-PCR kit (Cat: 11202ES03, YEASEN Biotech, Shanghai, China). Each reaction was also performed in triplicate. Relative RT–PCR was determined using ΔΔCT methods relative to control samples and internal control Gapdh. The primer sequence can be found in Supplementary Table 1.

### Enzyme Linked Immunosorbent Assay

Although the IgG or IgM detected by ELISA after heat-inactivation would be higher than those in the new plasma in patients diagnosed with COVID-19, the results were consistent with the positive rate of SARS-CoV-2 (Hu et al., 2020; Pastorino et al., 2020). Based on this, we analyze the mice antibody response by total IgM kit (Cat: 70-EK276-96, MULTI SCIENCES, Hangzhou, China) and SARS-Cov-2 spike receptor-binding domain (RBD) specific IgG kit (Cat: BD-PD266085, Biodragon, Suzhou, China) in serum. The standard process was provided as follows. First of all, the inactivated serum samples were diluted at a ratio of 1:100 with assay buffer. As the kit contains microplate strips with wells coated with corresponding proteins (IgM, anti-IgM, and SARS-CoV-2 spike receptor-binding domain antibody), the final 100 μl volume mix (1:200) containing 50 μl diluted serum and 50 μl assay buffer was incubated for 2 h in the wells. Then, a second incubation with horseradish peroxidase (HRP)-conjugated antibody was carried out for 1 h. After a proper color reaction, the OD value was calculated. According to kits instructions, the corrected values were saved for subsequent analysis.

### Multi-Cytokine Analysis

The cytokine was detected by LEGENDplex™ kit (Cat: 740007 and Cat: 740349, BioLegend, United States). The expression levels of cytokine and chemokines in serum were detected. According to the manufacturer’s protocol, all samples were diluted 2-fold, and the assay was performed in a V-bottom plate. Before the procedure, all the reagents were warm to room temperature (20–25°C), and the V-bottom plate was pre-washed by assay buffer twice. According to the recommended manual, the 25 μl plasma (after 2-fold diluted), 25 μl matrix, and 25 μl mixed beads were co-incubated at 800 rpm on a plate shaker for 2 h at room temperature. Then, the plate was centrifuged at 1,050 rpm (~250 g) for 5 min and immediately dumped the supernatant into a biohazard waste container by quickly inverting and flicking the plate in one continuous and forceful motion. Next, the plate was washed by dispensing 200 μl of Wash Buffer into each well and incubating for 1 min, and a second wash was conducted. Later, 25 μl of detection antibodies were added to each well, and the mixture was incubated at 800 rpm on a plate shaker for 1 h at room temperature. In the next step, 25 μl SA-PE were added to the co-incubated mix without washing the plate, and the mixture was shaken at 800 rpm on a plate shaker for 30 min. Subsequently, the procedures, including centrifugation and wash, were repeated. All incubation steps were placed in the dark or wrapped with aluminum foil. Finally, data were acquired by the Cytoflex flow cytometer (Beckman Coulter, Krefeld, Germany) and analyzed by the LEGENDplex™ Data Analysis Software (BioLegend, United States).

### Fluorescence *in situ* Detection

Fluorescence *in situ* detection (FISH) was performed for detecting the genomic RNA of SARS-CoV-2 virus from the fixed, paraffin-embedded (FFPE) tissues and followed the manufacturer’s protocol (Servicebio, Wuhan, China). The RNA probe oligonucleotides were synthesized by Servicebio and had been used to explore the SARS-CoV-2 infection on the placenta (Gao et al., 2021). The probe sequence was as follows: 5′-DIG-CCGTC TGCGG TATGT GGAAA GGTTA TGG-DI-3′. The FISH standard examination protocol had been described in the previous article (Gao et al., 2021). The infected lung was served as the positive control, while the uninfected lung was the negative control. Sections were counterstained with DAPI (Thermo Fisher Scientific), mounted, and stored at 4°C until image analysis. FISH images were captured on a lympus Eclipse 55i microscope (Olympus, Tokyo, Japan) and processed using ImageJ.

### Flow Cytometry

Expressions of cell surface molecules were evaluated by flow cytometry (FCM). PE-conjugated anti-mouse CX3CR1 antibody (Cat: 149005, BioLegend, United States), PE/Dazzle™ conjugated anti-mouse CD69 antibody (Cat: 104535, BioLegend, United States), APC-conjugated anti-mouse KLRG1 antibody (Cat: 138411, BioLegend, United States), AF700 conjugated anti-mouse TCR-β antibody (Cat: 109224, BioLegend, United States), BV421-conjugated anti-mouse CD44 antibody (Cat: 103040, BioLegend, United States), BV605-conjugated anti-mouse CD4 antibody (Cat: 100451, BioLegend, United States), BV605-conjugated anti-mouse CXCR3 antibody (Cat: 155915, BioLegend, United States), BV785-conjugated anti-mouse CD8 antibody (Cat: 100750, BioLegend, United States) were used. Here, the antibodies were incubated with peripheral blood mononuclear cells (PBMCs) acquired from 50 μl peripheral blood for 30 min before lysing the erythrocyte. The usages of antibodies were according to the instructions of the reagents. After incubation, 4% paraformaldehyde was used to fix cells and inactive viruses. Then, lysing erythrocyte was performed. Finally, the cells were resuspended in 100 μl of PBS for subsequent flow cytometric analysis (Beckman, Coulter, Krefeld, Germany). The FlowJo software was used to analyze the data acquired from FCM.

### Hematoxylin and Eosin

Organs were harvested from infected and uninfected mice and fixed with 4% paraformaldehyde, paraffin embedding and sectioning were performed. About 3–5 μm thick sections were stained with HE to find suspicious pathological changes caused by SARS-CoV-2 infection. The standard protocol of HE was performed by Biossci (Shanghai, China), which included dewaxing, staining with hematoxylin and eosin, mounting. The tissue sections were observed under an optical microscope. Furthermore, the results had been reviewed by more than two pathologists.

### Statistical Analysis

Wilcoxon and student *t*-test were used to analyze the significant difference between the two groups. Two-way ANOVA analyzed the time-related data with Bonferroni post-*t*-tests. All data were using GraphPad Prism Version 8 (San Diego, CA, United States) for data analysis. For all statistical tests, values of *p* < 0.05 were considered statistically significant.

## Results

### Pregnant Mouse Model of SARS-CoV-2 Infection and Pathogenesis

Angiotensin-converting enzyme II was the main membrane protein receptor that helped the SARS-CoV-2 enter the host cell (Benton et al., 2020). The mice engineered to hACE2 were widely used to study SARS-CoV-2 related *in vivo* research (Sia et al., 2020; Sun et al., 2020; Zheng et al., 2021). Here, the hACE2 expression in our target tissues (lung and uterus) in the transgenic mice had been verified by RT-PCR (Supplementary Figures S1A,B). It provided the important molecular basis for SARS-CoV-2 recognition and infection. To investigate whether the SARS-CoV-2 would establish infection in pregnant mice or cause adverse pregnancy outcomes, we inoculated the plugged transgenic hACE2- Chimera mice *via* the intranasal route with 5 × 10^3^ plaque-forming units (PFU) of SARS-CoV-2. The changes in body mass were monitored daily, and the mice were sacrificed at 4- or 7-dpi, as illustrated in Figure 1A. RT-PCR was used to detect the viral RNA in lung tissues. Both non-pregnant and pregnant mice showed a significant elevation of virus loads after infection. However, there was no difference between the groups of non-pregnant and pregnant at 4 dpi (*p* > 0.05; Figure 1B). Notably, the virus loads were significantly declined in the lungs of the non-pregnant mice (*p* < 0.05) at 7 dpi, which stayed high in the pregnant mice (*p* > 0.05). The results indicated that both pregnant and non-pregnant mice were susceptible to SARS-CoV-2 infection, while the pregnant mice displayed weaker virus removal ability. The histopathological changes were also obvious in the infected pregnant mice. There were considerable inflammatory cell infiltration, alveolar structural disorder, and fibrosis formation in the infected lungs at 4 dpi (Figure 1C, right upper) and 7 dpi (Figure 1C, right lower) compared with mock control (Figure 1C, left, upper for 4 dpi and lower for 7 dpi).

By comparing the weight change daily after infection, the infected mice displayed an evidential body mass reduction than uninfected control (Figure 1D). For infected pregnant mice, the weight loss was compromised by the fetuses’ weight, which increased along the gestational period. As shown in Figures 1E–G, the pregnant mice showed weight loss at first 2 dpi during the early pregnancy stage [started from embryonic day (E) 3.5 ± 1], while the weight of pregnant mice during early to middle (started from E7.5 ± 1) and middle to the late gestational stage (started from E12.5 ± 1) increased from the beginning (Figures 1F,G). Nonetheless, the proportions of weight changes were notably lower in the infected pregnant mice than in uninfected control (*p* < 0.05), indicating that infected pregnant mice showed significant weight loss.

### Outcomes of Pregnancy After SARS-CoV-2 Infection

**Figure 2:**
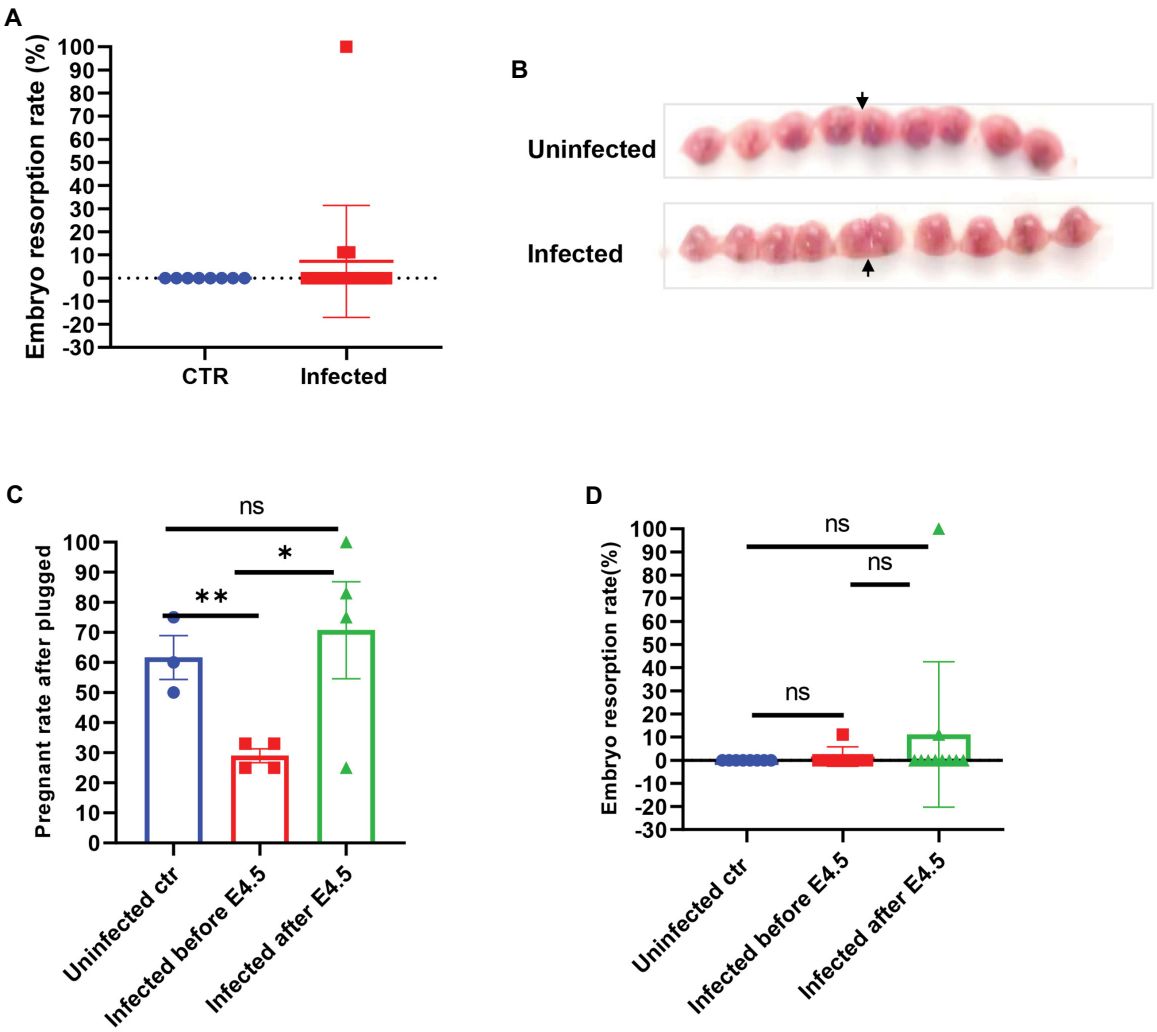
The embryos of SARS-CoV-2 infection in pregnant mice. **(A)** Embryo resorption rates between and uninfected control (CTR, *n* = 8) and infected pregnant (*n* = 17). **(B)** The typical graph of embryo development between uninfected (upper, E14.5) and infected (lower, E13.5). The site that the arrow pointed at was the uterine horn. **(C)** Further splitting the pregnant group into two groups by E4.5 (infected before E 4.5, *n* = 7 vs. infected after E 4.5 *n* = 10), the real pregnant rates after plugged (the numbers of pregnant mice/the numbers of plugged per experiment) were displayed. **(D)** Based on the prior group, the embryo resorption rates were compared. ns *p* > 0.05, ^*^p < 0.05, and ^**^p < 0.01.

The mated and plugged mice were recorded for their actual pregnancy state to establish whether the SARS-CoV-2 infection interfered with pregnancy, and we compared the development and absorption of embryos between infected and uninfected pregnant mice. There was no significant difference in the embryo resorption rate between the two groups (*p* > 0.05) and fetal development, as shown in Figures 2A,B. However, we noticed that if the plugged mice were infected before E4.5, which is the same time window of embryo implantation after plugged, the real pregnant rate would be much lower than that of other groups (uninfected control, 55.50–67.50%; infected before E 4.5, 25.00–33.00%; infected after E4.5, 62.50–87.25%, *p* < 0.05; Figure 2C). Besides, 100% resorption only occurred in one of the pregnant mice (Figure 2D). The fetuses were shown in Supplementary Figure S2A. However, no virus infection was found in the placenta (Supplementary Figure S2B). It was speculated that embryo absorption could be due to the intrauterine inflammatory changes caused by cytokine release syndrome after infection. We speculated that SARS-CoV-2 might induce implantation failure by targeting the embryo blastocytes and further analyzed the gene expression profile of receptors that mediate the SARS-CoV-2 virus infection from the opened RNAseq database. By analyzing the published RNAseq dataset, it was shown that pre-implantation embryos, the blastocysts of mice, expressed minimal levels of ACE2, while other genes, such as BSG (CD147) and TMPRSS2 (Jackson et al., 2021), expressed relative higher levels (Supplementary Figure S3A).

**Figure 3:**
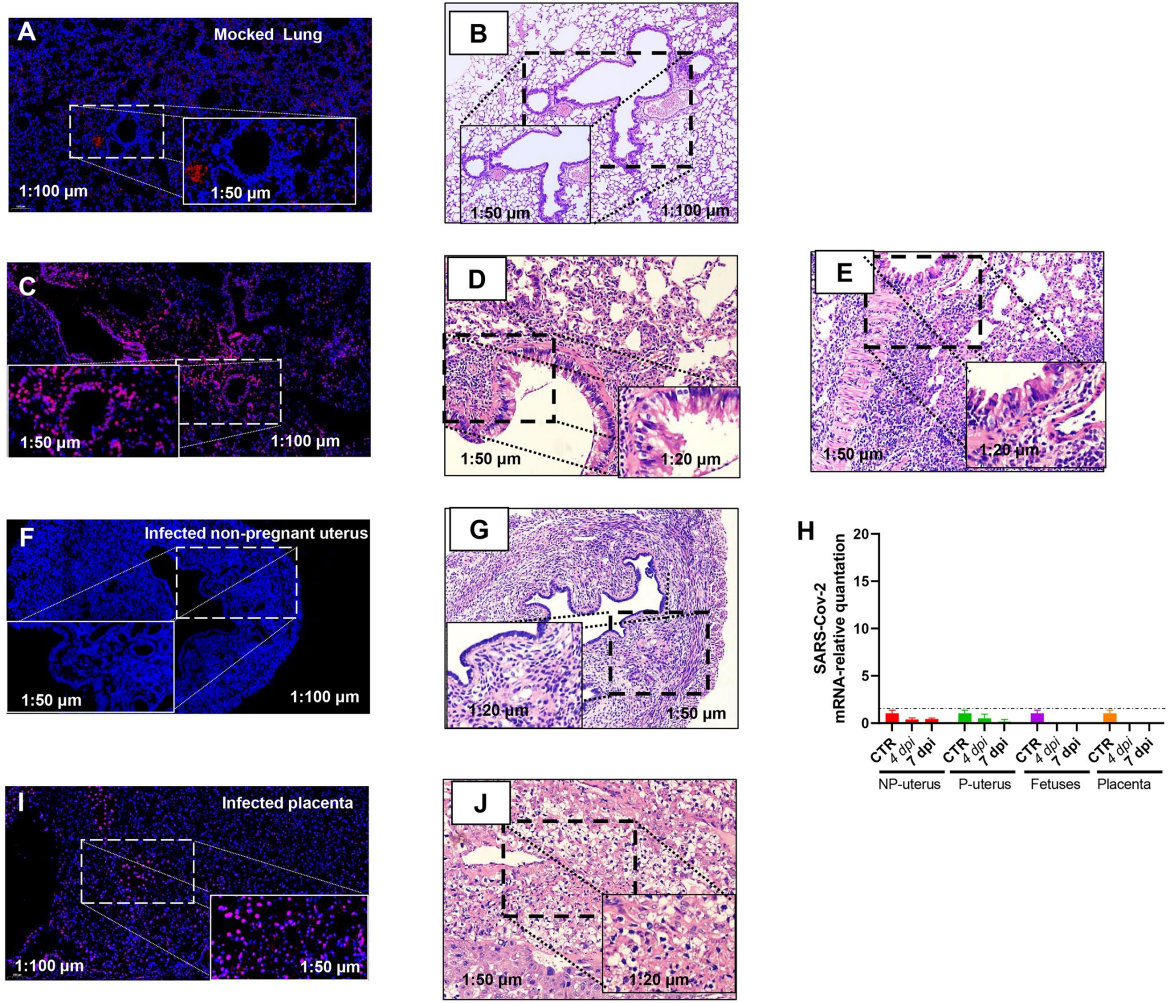
Fluorescence *in situ* hybridization (FISH) detection of SARS-CoV-2 mRNA. Mock lung **(A)** and corresponding HE **(B)**. Infected lung **(C)** and corresponding HE from the non-pregnant **(D)** and pregnant **(E)** mouse. Infected non-pregnant uterus **(F)** and corresponding HE **(G)**. **(H)** The SARS-CoV-2 mRNA relative quantitation of the uterus of non-pregnancy (*n* = 6) and pregnancy (*n* = 6), fetuses (*n* = 3), and placenta (*n* = 3) in infected mice. The SARS-CoV-2 RNA in fetuses and placenta had not been detected. NP, non-pregnancy; P, pregnancy; and Infected placenta **(I)** and corresponding HE **(J)**.

Otherwise, the intrauterine infection might also disturb the embryo infection. Thus, we evaluated the SARS-CoV-2 infection of the uterus by FISH. We verified the specificity of RNA-FISH using uninfected lung tissues (Figures 3A,B). The positive staining was distributed in the cytoplasm of lung cells (Figure 3C). It is in line with the virus distribution. The corresponding pathologic changes in the uninfected lung (Figure 3B) or infected lungs of non-pregnant (Figure 3D) or pregnant (Figure 3E) mice further supported the FISH staining result (both were from the lung of 7 dpi). These data suggested that the probes were effective in detecting SARS-CoV-2.

Nonetheless, viral RNA was not detected in infected non-pregnant female mice uterus (Figure 3F). The histopathology in the uterus of the non-pregnant pregnant mice was not changed (Figure 3G), which was consistent with the absence of viral RNA in the uterus by RT-PCR (Figure 3H). However, there was positive staining of FISH in the placenta of pregnant mice (Figure 3I), and the placenta displayed hyaline changes (Figure 3J), which was consistent with reports of SARS-CoV-2 detected in the human placenta of COVID-19 (Bouachba et al., 2021), although we had not detected SARS-CoV-2 RNA in placenta and fetuses by RT-PCR (Figure 3H). As the immune cell deposition in the lung (Figure 3E), the pregnant mice showed more severeness than that in non-pregnant mice (Figure 3D), we thus would like to explore further the differences in immune-responses to SARS-CoV-2 infection between pregnant and non-pregnant mice.

### Features of Proinflammatory Immune Responses

**Figure 4:**
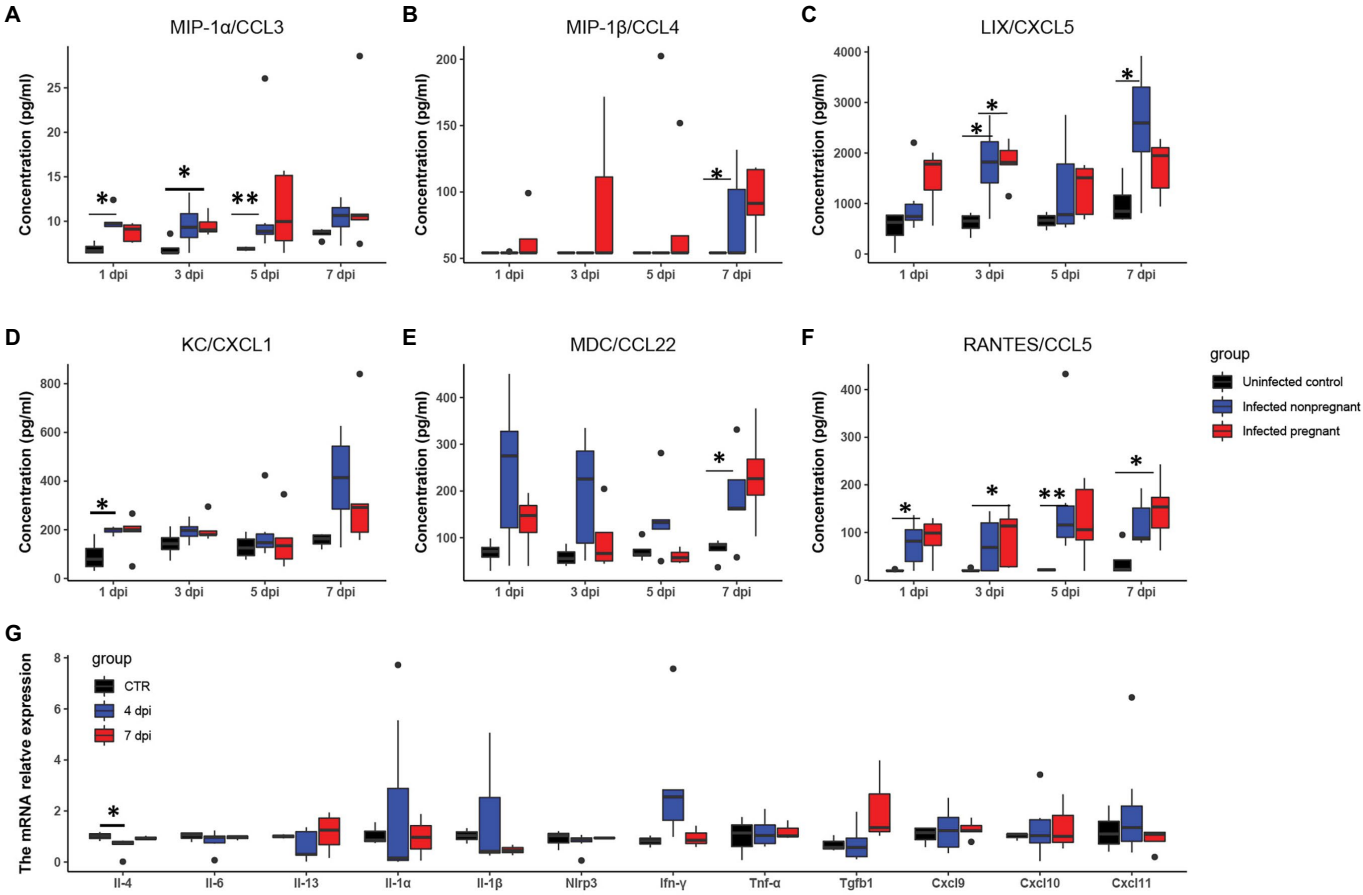
The feature of proinflammatory cytokines. The concentrations of “cytokine storm” related chemokines in plasma were measured. **(A–F)** Showed the changes of six chemokines (*n* ≥ 3 per each group). **(A)** MIP-1α/CCL3. **(B)** MIP-1β/CCL4. **(C)** LIX/CXCL5. **(D)** KC/CXCL1. **(E)** MDC/CCL22. **(F)** RANTES/CCL5. **(G)** The changes of pro-inflammatory cytokines in the uterus of infected non-pregnant mice. ^*^p < 0.05. ^**^p < 0.01.

To further explore the differences in the immune response between pregnant and non-pregnant mice after SARS-CoV-2 infection, we examined the dynamics of proinflammatory cytokine expression in the serum of peripheral blood in infected mice and displayed in the boxplots. The inflammatory cytokines, such as macrophage inflammatory protein-1 α/β [MIP-1α/β, MIP-1α, also known as chemokine (C-C motif) ligand 3 (CCL3); MIP-1β, also known as CCL4], keratinocyte-derived chemokine [KC, also known as chemokine (C-X-C motif) ligand 1 (CXCL1)], LPS-induced CXC chemokine (LIX, also known as CXCL5), regulated on activation normal T-cell expressed and secreted (RANTES, also known as CCL5), and macrophage-derived chemokine (MDC, also known as CCL22) were increased in the serum of mice after SARS-CoV-2 infection (Figures 4A–F). MIP-1α/β could enhance the monocytes’ chemotaxis and boost the inflammatory response (Menten et al., 2002). They were both elevated after infection in pregnant mice (^*^p < 0.05, ^**^p < 0.01), while the expression of MIP-1α was relatively higher in non-pregnant mice (Figures 4A,B). Expression of LIX and KC was increased in pregnant and non-pregnant mice (Figures 4C,D). Overall levels of LIX in non-pregnant mice were relatively higher than in pregnant mice. CCL22 is highly expressed by terminally differentiated macrophages and monocyte-derived dendritic cells, participating in the recruitment of immature macrophages and monocytes (Godiska et al., 1997). It was highly produced in non-pregnant mice at 1–3 dpi, while major increased in pregnant mice at 7 dpi (Figure 4E). These changes indicated that infected mice elicited an effective innate immune response. The CCL5/CCR5 axis is a critical pathway involving disease progression, which recruits and activates T cells (Patterson et al., 2021). The expression levels of CCL5 were upregulated in the non-pregnant mice after SARS-CoV-2 infection at 1 and 5 dpi (^*^p < 0.05, ^**^p < 0.01), while its peak expression was delayed in pregnant mice mainly at 7 dpi (*p* < 0.05; Figure 4F).

It has been shown that a proper inflammatory environment is required for successful implantation. To determine whether cytokine storms disturbed the intrauterine cytokine homeostasis, we performed RT-PCR to examine several inflammatory cytokines changes in the non-pregnant uterus (Figure 4G). Most of the cytokines in the infected non-pregnant were equivalent to the mocked control. T, the expression levels of several cytokines such as Il-1α, Il-1β, and Ifn-γ showed a wide range variance. These results were consistent with the FISH of the non-pregnant uterus in that the virus copies were absent (Figure 3F). The inflammatory cytokines might indicate that uterus response to the immune system could be one of the reasons for the failure of implantation.

Furthermore, the production of inflammatory cytokines showed a distinct trend between pregnant and non-pregnant. Overall, these data demonstrated that mice infected by SARS-CoV-2 would activate the innate immune response and increase the secretion of inflammatory factors. In short, the infection may cause a slight disturbance of the local immune environment in the uterus.

### Lower T Cell Activation in Pregnant Mice

**Figure 5:**
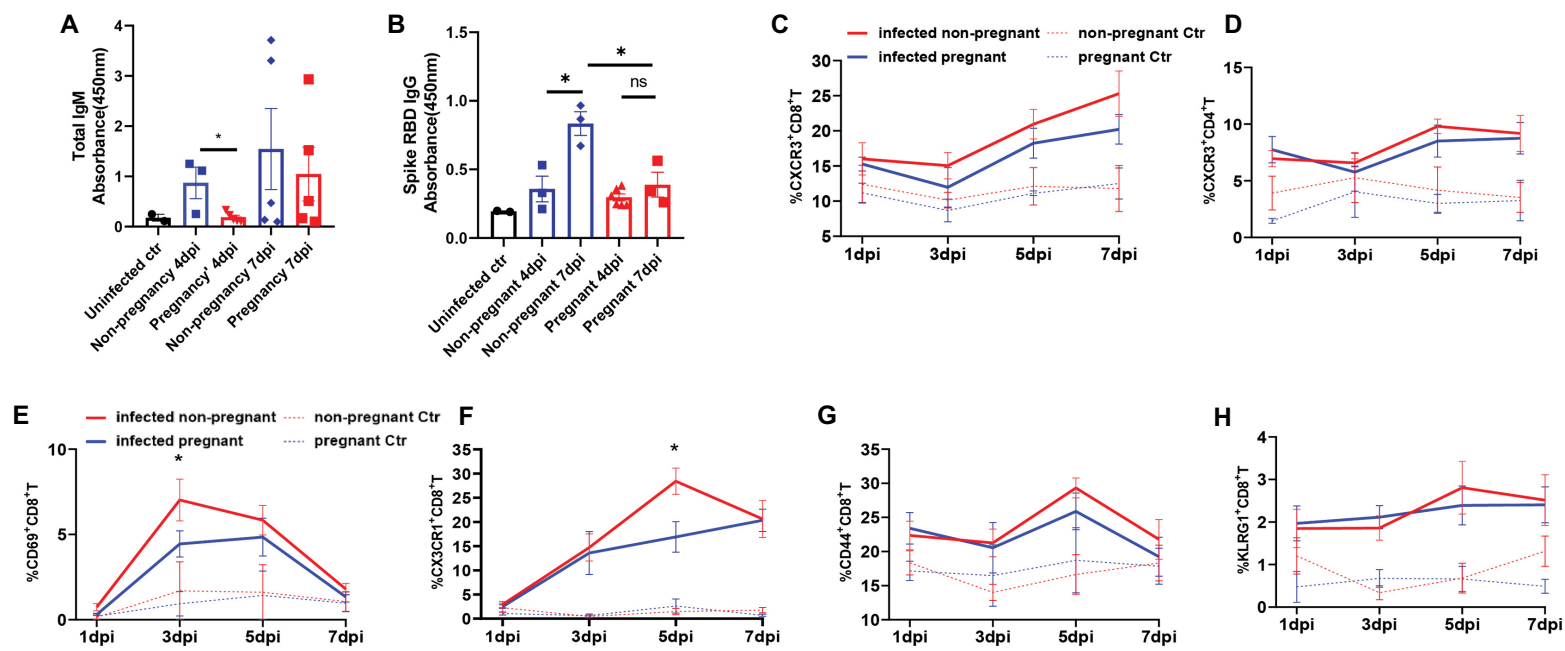
The production of antibodies and the activation of T cells. Total IgM **(A)** or SARS-CoV-2 spike receptor-binding domain (RBD) IgG **(B)** in plasma of mice was detected. The absorbance of OD 450 nm was compared between the pregnant and non-pregnant groups during 4–7 dpi (*n* ≥ 3 per each group, unpaired *t*-test, ^*^p < 0.05, ns *p* > 0.05). The gates of flow cytometry (FCM) are shown in Supplementary Figure 4. The T cell subsets at 1, 3, 5, and 7 dpi were analyzed by flow cytometry between infected non-pregnant mice (red line) and that of pregnant mice (blue line; **C–H**, *n* ≥ 3 per each group). **(C)** %CXCR3^+^CD8^+^T cells. **(D)** %CXCR3^+^CD4^+^T cells. **(E)** %CD69^+^CD8^+^T cells. **(F)** %CX3CR1^+^CD8^+^T cells. **(G)** %CD44^+^CD8^+^T cells. **(H)** % KLRG1^+^CD8^+^T cells. **(C–H)** Unpaired *t*-test and Welch’s *t*-test were used. ^*^p < 0.05, ns *p* > 0.05.

As adaptive immunity, mainly mediated by antibody and cytotoxic T cells, are vital in virus clearance, we then analyzed the antibody production and T cells activation from the infected mice. IgG and IgM are the most important antibodies that protect from pathogens attacking. We detected that the total IgM was secreted mildly in the serum of pregnant mice but was increased robustly in non-pregnant mice’s serum and further elevated at 7 dpi (Figure 5A). The levels of SARS-CoV-2 spike RBD specific IgG, were continuously elevated during 4–7 dpi in the infected non-pregnant group while remaining unchanged in pregnant mice (*p* > 0.05; Figure 5B).

Multi-channel flow cytometry was used to analyze the dynamic changes of different activation markers in T cells of peripheral blood from non-pregnant or pregnant mice at 1, 3, 5, and 7 dpi. The different activation or functional markers for T cells were screened, and the gating strategy was shown in Supplementary Figure S4. Overall, the T cells were significantly activated after infection compared with uninfected controls. Furthermore, there was no significant difference in the dynamics of proportions of CXCR3^+^CD4^+^T (Figure 5C), and CXCR3^+^CD8^+^T cells (Figure 5D) between pregnant and non-pregnant mice, although they were both elevated after infection. CD69 was an early activation marker for CD8^+^T cells, rapidly induced in mature T cells when stimulated by T cell antigen receptor (TCR; Yamashita et al., 1993). Furthermore, CX3CR1^+^CD8^+^T cells were recently identified as cytotoxic T cells (Böttcher et al., 2015; Gerlach et al., 2016; Chen et al., 2021b). In our results, the percent of CD69^+^CD8^+^T cells (Figure 5E) was significantly lower in pregnant mice than non-pregnant at 3 dpi, as well as CX3CR1^+^CD8^+^T cells (Figure 5F), which were considerably less at 5 dpi. It suggests that CD8^+^T cells’ activation or differentiation may be suppressed in pregnant mice after SARS-CoV-2 infection. Expressions of CD44 and KLRG1 molecules in CD8^+^T cells were elevated after virus infection (Figures 5G,H); however, there was no difference between non-pregnant and pregnant mice. In summary, the pregnant mice showed an inadequate anti-virus immune response of antibody production and T cells activation and differentiation.

## Discussion

Although some COVID-19 disease cases were reported for evidence of mother-to-fetal transmission at a terminal gestational stage, it is difficult to assess whether SARS-CoV-2 infection would interfere with gestation and whether the pregnancy would affect vulnerability and the immune response to SARS-CoV-2 infection. This study utilized mated hACE2 transgenic mice and illustrated distinct biological differences in how pregnant and non-pregnant mice respond to SARS-CoV-2, while the vertical transmission was absent in our model.

After intranasal SARS-CoV-2 inoculation, hACE2 pregnant mice showed mild weight “loss,” and high levels of viral RNA were detected in the lungs, which elicited elevated levels of proinflammatory cytokines and chemokines in the serum. The combined infection and inflammation resulted in apparent interstitial pneumonia characterized by collapsed alveolar spaces and lymphocyte infiltration in the lung parenchyma. The symptoms after SARS-CoV-2 infection in hACE2 pregnant mice are consistent with the subclinical or mild disease feature in most human cases.

In a normal pregnancy, the average gained weight over pregnancy is more than three times of unpregnant mice due to placenta and embryo development. Although the body mass changes of pregnant mice after infection were significantly decreased compared with the uninfected pregnant group, there was no significant difference in the embryo absorption rate. The embryonic development of infected mice is not significantly different from that of uninfected mice. Although the embryos from a pregnant mouse showed complete absorption, no virus infection was found in the placenta. It was speculated that embryo absorption would be due to multiple organ failure and systemic hypoxia caused by severe infection symptoms. In addition, no virus RNA was detected in the embryo by RT-PCR. It was consistent with previous literature reports for pregnant females. In summary, the virus infection did not influence the fetuses’ development, and the weight loss of pregnant mice can be mainly due to muscle wasting rather than pregnancy loss or pregnancy complications.

In our study, all the mice were transgenic mice, and almost 55.50–67.50% of uninfected mated mice were successfully pregnant. It was in line with published data (62.9%, 173/275, pregnant mice/mice with copulation plugs; Heyne et al., 2015). It indicated that the human ACE2 transgenic would not disrupt the pregnant. However, our data revealed a significant reduction (25.00–33.00%) in the real pregnancy rate of the mated female mice infected before the implantation. And the decrease in pregnancy rate did not result from directly infecting the endometrium because we had not detected the virus in the non-pregnant uterine tissues by RT-PCR or FISH. It was a surprising discovery. As a recent study showed ACE2 is a key molecule in the process of endometrial stromal cells (ESC) decidualization and the treatment with ACE2-targeting siRNAs would induce a failure decidualization (Chadchan et al., 2021), a critical hypothesis for our next research is whether the SARS-CoV-2 infection would downregulate the ACE2 expression and eventually lead to a failure decidualization.

Researchers had found that ZIKV can interfere with embryo implantation in mice by infecting the blastocyst ectoderm and cause implantation failure (Tan et al., 2019). Our analysis showed that SARS-CoV-2 might also directly infect mouse blastocysts by the receptor, BSG, and TMPRSS2, which were highly expressed in mice blastocysts, and cause implantation failure based on the open-accessed dataset (Tan et al., 2019). Currently, there was no report about human embryo implantation failure during the SARS-CoV-2 infection, more likely because it was difficult to observe in the clinical practices. Multiple articles reported that human blastocysts express ACE2, BSG, and other proteins that help SARS-CoV-2 enter the host cells (Chen et al., 2020; Colaco et al., 2021), indicating that SARS-CoV-2 might cause implantation failure by directly infecting the blastocysts in humans.

As reported, the pre-implantation embryo could induce a uterine inflammatory reaction in mice (Zhu et al., 2021), and successful implantation is associated with a transient increase in serum proinflammatory cytokine profile (Zhao et al., 2021). Together, it indicated that implantation accompanies an intricacy inflammatory regulatory net between the blastocysts and maternal uterus, even maternal immune system. Besides, implantation loss in female mice caused by intraperitoneal injection of lipopolysaccharide (LPS) indicates that inflammation changes can impair the inflammatory state of the uterus during the implantation window (Moustafa et al., 2019). In male mice, the SARS-CoV-2 infection could disrupt the blood-testis barrier through the induction of inflammatory cytokines and disruption of junctional protein rather than directly invading the target cells (Peirouvi et al., 2021). It means that the cytokine storm caused by SARS-CoV-2 infection could impair the peripheral organs and result in an interstitial inflammation. In present study, we observed elevated cytokine levels in the plasma of SARS-CoV-2 infected mice model. Besides, the uterine cytokines, such as Il-1α, Il-1β, and Ifn-γ in those non-pregnant mice display a wide range of fluctuations although there were no SARS-CoV-2 viruses in uterus. It indicated that the uterine immune environment was aroused by the peripheral cytokines storm. However, an uncontrolled uterine environment was not conducive to embryo implantation. Therefore, we postulated that the systemic inflammation induced by SARS-CoV-2 might be a major reason for implantation failure.

As the plasma was heat-inactivated, most cytokines’ concentration was factitious downregulated or even lower than the detection value (Xu et al., 2021), which may be the reason why our multi-factor detection kits detected a few cytokines. In our study, cytokines, such as MIP-1α/β, RANTES/CCL5, MDC/CCL22, KC/CXCL1, and LIX/CXCL5 were upregulated after infection. MIP-1α is significantly elevated in critically ill patients and can be used as a potential marker for predicting the severity of COVID-19 disease (Huang et al., 2020). The overall level of MIP-1α in non-pregnant mice and pregnant mice was elevated and there was a transient increase at 5 dpi in pregnant mice. It indicated that the prognosis of SARS-CoV-2 infection in pregnant mice might be poor.

After infection, the innate immune cells were first activated to secret chemokines to recruit and activate peripheral innate immune cells or adaptive immune cells. MDC, KC, and LIX are inflammatory factors secreted by macrophages, which promote the targeted enrichment and activation of peripheral innate immune cells (Mantovani et al., 2000; Kobayashi, 2008; Mohammadi and Kariminik, 2021). Our results showed that although the lung load of pregnant mice was equivalent to that of non-pregnant mice, the increase of plasma LIX, MDC, and other inflammatory cytokines was lower than that of non-pregnant mice. RANTES is an inflammatory factor that promotes cytotoxic cells such as NK cells and T cells (Kohlmeier et al., 2008; Maghazachi, 2010; Tamamis and Floudas, 2014). The increase of RANTES in pregnant mice has mainly distributed in 5 and 7 dpi, which may be one of the reasons for the insufficient increase in the proportion of CD69^+^CD8^+^ T cells in pregnant mice. In summary, the cytokines changes in pregnant mice were consistent with the T cells proliferation and activation, and the increase of cytokines in pregnant mice was later than those in non-pregnant.

We found that virus clearance, antibody production, and T cell activation in pregnant mice were weaker than in non-pregnant mice. It might be a special performance during pregnancy. Progesterone is an important reproductive endocrine hormone for maintaining pregnancy during pregnancy. According to reports, progesterone has a pivotal immunomodulatory property (Druckmann and Druckmann, 2005). Progesterone can inhibit the innate immune response of macrophages by inhibiting the activation of NF-κB (Su et al., 2009) and CD8^+^ T cell viral-specific effector function (Cherpes et al., 2008). In mouse models of influenza A virus infection, treatment with progesterone or progesterone and levonorgestrel also reduced virus-specific antibodies and virus-specific levels (Hall et al., 2017). The high progesterone level might be the reason for the limited production of specific IgG and activation of CX3CR1^+^CD8^+^T cells in the pregnant mice after infection, which needed further experimental confirmation. Moreover, insufficient present antigen information dominated by upregulation progesterone may be another hypothesis for lower CD8^+^ T cells activation.

However, whether the decidual stromal cells (DSCs) play a role in inhibiting the activation of CD8^+^ T cells should be considered. It has been reported that the DSCs could partly derive from bone marrow, and pregnancy could stimulate bone marrow derived cells into the uterus (Tal et al., 2019). And administering DSCs by infusion could alleviate the cytokine storm in SARS-CoV-2 patients emphasizing the immunosuppressive effect (Sadeghi et al., 2021). Besides, we had found that the DSCs could modulate CD8^+^ T cells and exhibit more tolerance (Liu et al., 2020). Together, whether the DSCs could secret cytokines or hormones to inhibit the CD8^+^ T cells activation should be taken into consideration, especially in the condition of SARS-CoV-2 infection. And whether the migrated bone marrow derived cells would inhibit the activation of CD8^+^ T cells was another interesting hypothesis.

Researchers had found that the increase in plasma cytokines in non-pregnant women in humans was significantly higher than that in the pregnant, which was consistent with our results, and there was no considerable difference in the ratio of total T cells, CD4^+^, T cells, CD8^+^ T cells, and the ratio of CD4^+^/CD8^+^ in peripheral blood mononuclear cells (Chen et al., 2021a). In our study, there was no significant difference in the number of CXCR3^+^CD8^+^T cells and CXCR3^+^CD4^+^T, CD44^+^CD8^+^T, and KLRG1^+^CD8^+^T between pregnant and non-pregnant mice, but the proportion of CD69^+^CD8^+^ T cells, increasing mainly at the early infection, and CX3CR1^+^ CD8^+^ T cells acted as an antiviral defender, was significantly downregulated, which emphasizes the deficiency of pregnancy in defending viral infections in females.

The human placenta differs in morphology from the mouse placenta but shares some common features. Both placentas are classified as hemochorial type, which means that the fetal trophoblast tissue is directly immersed in the maternal blood (Furukawa et al., 2014). SARS-CoV-2 virus is mainly translocated through the blood vessels to peripheral organs. That is to say, the mice model could mimic the pathogenic characteristics of SARS-CoV-2 on the uterus or placenta and could partly interpret how the virus invades human beings. However, considering that the human placenta is composed of one syncytial layer while the mouse placenta consists of three layers: the labyrinth, the spongiotrophoblast, and the maternal decidua, and the mouse exhibits shallow intrauterine trophoblast invasion, whereas the human possesses deep intrauterine trophoblast invasion (Soares et al., 2017). Whether SARS-CoV-2 infection on human pregnant women should be paid more attention for the report that the SARS-CoV-2 could invade the trophoblasts (Debelenko et al., 2021).

The present study found that SARS-CoV-2 infection can decrease embryo implantation rate; however, our study did not prove how SARS-CoV-2 induces implantation failure. Our results were still inadequate. Due to the research limitations on dangerous infectious diseases such as SARS-CoV-2, all samples need to be inactivated before laboratory examining, resulting in unsatisfactory results for our inflammatory factors. Mouse pregnancy featured as generally multiple pregnancies simultaneously, which was different from a human singleton pregnancy. The selection during collecting placenta samples from mice may affect the final experimental results. In our study, positive staining of SARS-CoV-2 was detected in FISH samples. However, it was negative in RT-PCR. Considering the consistent results of FISH and RT-PCR in the detection of lung samples, we had ruled out the possibility of experimental errors. We deduced that in multiple pregnancies in mice, some but not all of the placenta may be infected by SARS-CoV-2 infection, leading to the different results of our different experiments.

In summary, we found the differences in virus elimination after the infection of SARS-CoV-2 in pregnant and non-pregnant mice, which correlated with different levels of antibody production and T cell activation. Our data provide information for understanding the effect of pregnancy on the pathogenesis of SARS-CoV-2 infection, which may aid in optimizing clinical management and eventually better understand SARS-CoV-2 biology as we are confronted with emerging variants and preventing neonatal outcomes infection.

## Data Availability Statement

The original contributions presented in the study are included in the article/Supplementary Material; further inquiries can be directed to the corresponding authors.

## Ethics Statement

The animal study was reviewed and approved by Animal Ethics Committee of Shanghai Medical College of Fudan University.

## Author Contributions

MD, LL, and QC contributed to the conception and design of the study. GZ, SD, and LL performed the statistical analysis and wrote the sections of the manuscript. GZ, SD, LL, GH, and YW performed the experiments. XH and GH helped with the figures. XL, DL, YZ, and DQ revised the whole manuscript. All authors contributed to the article and approved the submitted version.

## Funding

This work was supported by the grants from the National Natural Science Foundation of China (31970859, 81630036, 91542116, and 82101706), the International cooperation project between Macau and Shanghai (20410760300), an Innovative research team of high-level local universities in Shanghai, Shanghai Sailing Program (21YF1403700), Shanghai Municipal Health and Family Planning Commission (20204Y0403), National Key Development and Research Program (2021YFA1300803), and a key laboratory program of the Education Commission of Shanghai Municipality (ZDSYS14005).

## Conflict of Interest

The authors declare that the research was conducted in the absence of any commercial or financial relationships that could be construed as a potential conflict of interest.

## Publisher’s Note

All claims expressed in this article are solely those of the authors and do not necessarily represent those of their affiliated organizations, or those of the publisher, the editors and the reviewers. Any product that may be evaluated in this article, or claim that may be made by its manufacturer, is not guaranteed or endorsed by the publisher.
