# Supplementary material for: Delayed Antiviral Immune Responses in Severe Acute Respiratory Syndrome Coronavirus Infected Pregnant Mice
Source: Front Microbiol. 2022 Jan 21;12:806902. doi: 10.3389/fmicb.2021.806902 (PMC8814454; doi:10.3389/fmicb.2021.806902)
Supplement: Supplementary file 1 [file Data_Sheet_1.pdf]

Supplementary Material

1 Supplementary Figures and Tables

1.1 Supplementary Figures

Supplementary figure 1. The expression of hACE2 in transgenic mice. (A) The mRNA qualification of human ACE2 was detected in transgenic hACE2 mice by RT-PCR. Ute: uterus; WT: wild type. (B) Agarose gel electrophoresis for hACE2 PCR production.

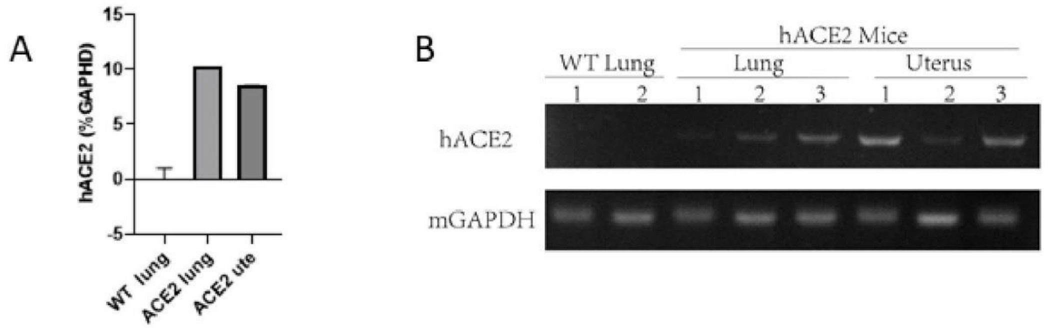

Supplementary figure 2. The complete absorption fetuses. (A) The complete absorption of embryos from the infected mice (E 17.5). (B) The FISH of the placenta from the absorption embryos.

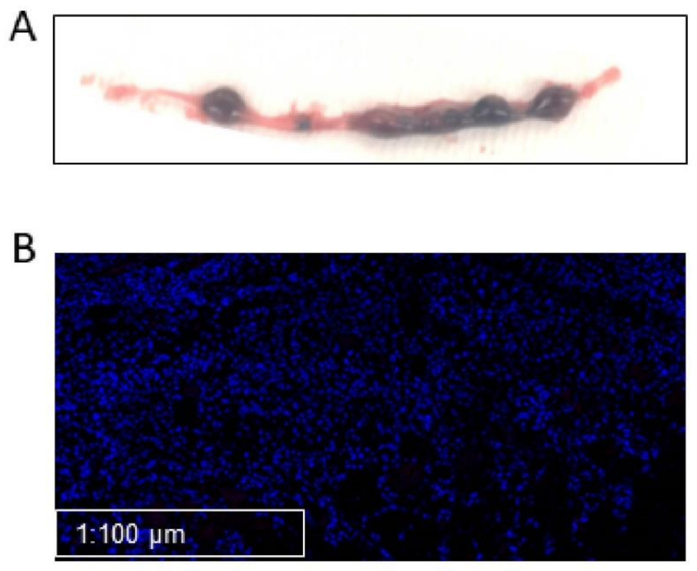

Supplementary figure 3. (A) The expression level of several proteins correlated with SARS-CoV-2 entering the target cell in mice blastocyst. Data was acquired from the Gene Expression Omnibus Series (GSE) 133254 mock group. Receptor: ACE2, BSG, AXL, DPP4, KREMEN1, ASRGL1. Coreceptor: NRP1. Cofactor: TMPRSS2, FURIN.

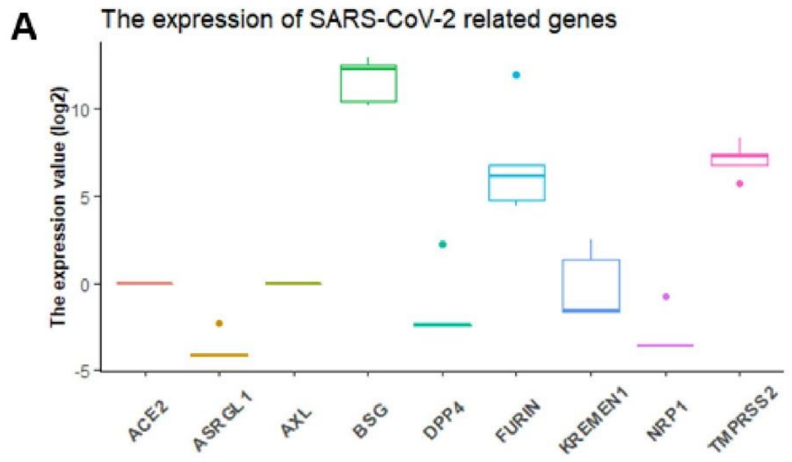

Supplementary figure 4. The gating strategy for Flow cytometry.

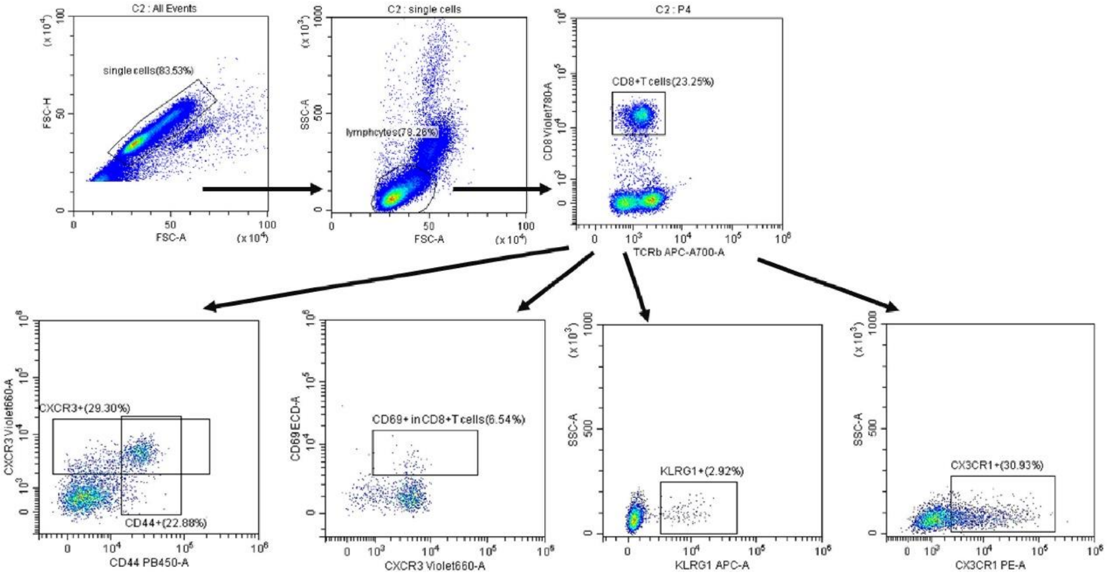

1.2 Supplementary Table

The primer sequence is shown below.

Gene		Sequence (5'-3')
hACE2	Forward	TGCAGCCACACCTAAGCATT
	Reverse	GTCACATTTGTGCAGAGGGC
SARS-CoV-2 N	Forward	GGGGAACTTCTCCTGCTAGAAT
	Reverse	CAGACATTTTGCTCTCAAGCTG
	Probe	TTGCTGCTGCTTGACAGATT
Gapdh	Forward	CAATGTGTCCGTCGTGGATCT
	Reverse	GTCCTCAGTGTAGCCCAAGAT
	Probe	CGTGCCGCCTGGAGAAACCTGCC
Il-4	Forward	GGTCTCAACCCCCAGCTAGT
	Reverse	GCCGATGATCTCTCTCAAGTGAT
Il-6	Forward	ATCCAGTTGCCTTCTTGGGACTGA
	Reverse	TAAGCCTCCGACTTGTGAAGTGGT
Il-13	Forward	GTATGGAGTGTGGACCTGGC
	Reverse	TTTTGGTATCGGGGAGGCTG
Il-1α	Forward	CGCTTGAGTCGGCAAAGAAA
	Reverse	CTTCCCGTTGCTTGACGTTG

Il-1β	Forward	AATGCCACCTTTTGACAGTGATG
	Reverse	AGCTTCTCCACAGCCACAAT
Nlrp3	Forward	ATTACCCGCCCGAGAAAGG
	Reverse	TCGCAGCAAAGATCCACACAG
Ifnγ	Forward	GCTACACACTGCATCTTGGC
	Reverse	CATGTCACCATCCTTTTGCCAG
Tnf-α	Forward	AGGGTCTGGGCCATAGAACT
	Reverse	CCACCACGCTCTTCTGTCTAC
Tgfb1	Forward	CTGGAGTTGTACGGCAGTGG
	Reverse	GGTTCATGTCATGGATGGTGC
Cxcl9	Forward	TGTGGAGTTCGAGGAACCCT
	Reverse	GCCTCGGCTGGTGCTG
Cxcl0	Forward	CCCACGTGTTGAGATCATTG
	Reverse	TCCATCACAGCACCGGG
Cxcl11	Forward	GCGACAAAGTTGAAGTGATTG
	Reverse	GCATGTTCCAAGACAGCAGA
